# Supplementary material for: A high-resolution mRNA expression time course of embryonic development in zebrafish
Source: eLife. 2017 Nov 16;6:e30860. doi: 10.7554/eLife.30860 (PMC5690287; doi:10.7554/eLife.30860)
Supplement: Supplementary file 6. [file elife-30860-supp6.zip › biolayout-clusters-files/Cluster190.html]

Cluster190


# Cluster190: Detail

### Go to ZFA detail

## GO

| GO ID | Description | Domain | Annotated | Expected | Observed | Adjusted p-value | Genes | Ensembl IDs |
| --- | --- | --- | --- | --- | --- | --- | --- | --- |
| GO:0043648 | dicarboxylic acid metabolic process | biological\_process | 19 | 0.01 | 3 | 4.6e-05 | mdh1aa mdh2 fh | ENSDARG00000017772 ENSDARG00000043371 ENSDARG00000075132 |
| GO:0006099 | tricarboxylic acid cycle | biological\_process | 12 | 0.01 | 3 | 1.0e-05 | mdh1aa mdh2 fh | ENSDARG00000017772 ENSDARG00000043371 ENSDARG00000075132 |
